# TLR-3 and TLR-7/8 ligands indirectly activate Langerhans cells when intradermally injected by triggering the recruitment of inflammatory cells

**DOI:** 10.1186/1742-4690-9-S2-P7

**Published:** 2012-09-13

**Authors:** O Epaulard, L Adam, R Le Grand, F Martinon

**Affiliations:** 1 CEA/Division of Immuno-Virology, Fontenay aux Roses, France

## Background

TLR-3 and TLR-7/8 agonists are promising vaccine adjuvants but their effects on immune cells remain to be define in vivo. We analysed the modifications in the Langerhans cell (LC) network after intradermal injection of these TLR ligands in non-human primates used as a model for assessing human vaccines. LCs, which are the only antigen-presenting cells in the epidermis, are endowed with great ability to induce immune responses.

## Methods

Intradermal injection of poly(I:C), R848 or PBS in cynomolgus macaques was followed by biopsy of the injection site at different time points. Epidermal and dermal sheets were dissociated, and epidermal and dermal cells were extracted before flow cytometry and culture. In situ immunofluorescence was performed on snap-frozen skin biopsies. Macaque polymorphonuclear neutrophils (PMNs) and monocytes were isolated from blood.

## Results

Cynomolgus macaque epidermis contains CD45+, DR+, CD1a, CD207+ LC, and dermis contains DR+, CD11c+ dermal dendritic cells (featuring CD1a + CD14- dermal DCs and CD1a- CD14+ DCs) and DR+, CD11c-, CD163+ macrophages. R848 intradermal injection results in LC maturation and activation (increased expression of CD80, CD83 and CD86) and migration (in situ immunofluorescence) out of the skin. PMNs and macrophages were massively recruited locally (up to 17% and 20% of total leucocytes, respectively, whereas steady state values are below 3%); PMN recruitment and LC activation were significantly correlated suggesting the role of R848 induced inflammation on LC changes and migration. Similar results, with lower intensity, were obtained after poly(I:C) injections. The involvement of inflammatory cells was confirmed by the lack of TLR-7/8 expression in LCs and the effect of secreted cytokines.

## Conclusion

Better understanding of the dynamics of local inflammation induced by TLR ligands in relevant animal models is critical for improving human vaccine adjuvants.

